# Hepatitis C hospitalizations in Spain, 2004-2013: a retrospective epidemiological study

**DOI:** 10.1186/s12913-017-2410-1

**Published:** 2017-07-05

**Authors:** R. Boix, R. Cano, P. Gallego, F. Vallejo, R. Fernández-Cuenca, I. Noguer, A. Larrauri

**Affiliations:** 1 National Centre of Epidemiology, Institute of Health Carlos III, C/Monforte de Lemos, 5, 28029 Madrid, Spain; 2 CIBER Epidemiología y Salud Pública (CIBERESP), C/ Monforte de Lemos, 3-5, 28029 Madrid, Spain

**Keywords:** Hepatitis C, Hospitalizations, Trends, Epidemiology

## Abstract

**Background:**

Hepatitis C is an important public health problem about which there is currently scarce epidemiological information. The objective of this study is to describe and analyse the demographic and epidemiological characteristics of hospitalized cases of hepatitis C in the Spanish population between 2004 and 2013.

**Methods:**

The study uses the Hospital Discharge Records Database of the Spanish National Health System. It is a retrospective descriptive epidemiological study. The variables analysed were year of infection, age, sex, diagnostic category, days admitted and co-morbidity.

**Results:**

There have been a total of 351,996 hospitalizations; 225,138 men (64%) and 126,858 women (36%). They are divided between acute hepatitis 8161 (2.3%); chronic hepatitis 325,185 (92.4%) and unspecified hepatitis 18,650 (5.3%). The mean age for men is 53.7 (+/−15.2) and for women 62.3 (+/−17.3). 22.8% also present with an Human immunodeficiency virus (HIV) disease coinfection, and 14.7% with opioid dependencies. The trend is for a gradual increase in cases without statistical significance.

**Conclusions:**

The Hepatitis C cases hospitalized had high levels of chronicity, which entails two distinct patterns of illness in men and women – who are affected in different age ranges.

## Background

Despite the fact that hepatitis C is a disease of particular relevance to public health, the epidemiological information available remains scarce. This, in part, is due to the intrinsic characteristics of a disease which is difficult to diagnose, remains asymptomatic for many years and becomes chronic in a high percentage of cases. It is estimated that hepatitis C affects 150 million people globally, of which 350,000 to 500,000 die each year as a result. In the World Health Organization (WHO) European Region it is estimated that there are 15 million people infected [1]. In 2013 the European Centre for Disease Prevention and Control (ECDC) was notified of 31,513 cases of hepatitis C in 26 countries [2], of which 569 (1.8%) were acute, 4776 (15.2%) were chronic, and 23,230 (73.7%) were unknown. A number of studies have been undertaken in Spain to evaluate the prevalence of hepatitis C [3–13], in general they are reviews [3] or local-level studies, carried out in both distinct populations and the general population [4–8] (with a range of prevalence between 0.74% and 2.6%), school-age children [9] (0.36%), pregnant women [10–12] (0.4-1.4%) and the working population [13] (0.6%).

In the recently approved Strategic Plan for Tackling Hepatitis C in the Spanish National Health System [14], it is estimated that 1.7% of the Spanish adult population (688000) have antibodies against hepatitis C and 1.2% of adults have viremia (472000) [15, 16].

In addition to studies on the prevalence, other studies of mortality and disease burden show that when the chronic complications of hepatitis C – like cirrhosis and hepatocellular carcinoma – are taken into account, Hepatitis C leads the list of infectious disease related mortality in Spain [17] and is the leading cause of disability-adjusted-life-years (DALYs) among transmissible diseases in Spain [18].

The Hospital Discharge Records Database of the Spanish National Health System (CMBD in its Spanish acronym) registers the cases in National Health Service hospitals, as well as a number of private hospitals, with a 90% level of coverage at present [19]. The system collects information regarding the patient, the disease and the hospital. The information system based on the CMBD can be a useful tool to better understand the epidemiological pattern of the disease, and the associated assistance required.

The objective of this study is to describe and analyse the demographic and epidemiological characteristics of those cases hospitalized with a diagnosis of hepatitis C and in addition to identifying trends in the hospitalizations. The study uses the CMBD database as an information system.

## Methods

This is a descriptive retrospective epidemiological study of the hospitalizations with hepatitis C diagnoses in the Spanish population from 2004 to 2013.

It has used data from the CMBD, which have been coded with the International Classification of Diseases, Ninth Revision, Clinical Modification (ICD-9-CM) from the World Health Organisation [20].

CMBD is the Spanish mandatory hospital discharge registry. Similar registries are in place in western countries. In Spain the registry uses the ICD-9 CM for codifying the illnesses and in 2017 will begin to use the International Classification of Diseases, Tenth Revision (ICD-10). The registry does not permit distinguishing between hospitalization and patients. CMBD has been consolidated as the principal source for knowing about the causes of disease which receive treatment in hospitals. Its application to epidemiological studies is shown by many scientific articles [21–23].

It has studied all of those hospital admissions in which a primary or secondary diagnosis with one of the following codes appeared: 070.41 (acute hepatitis C with hepatic coma), 070.44 (chronic hepatitis C with hepatic coma), 070.51 (acute hepatitis C without mention of hepatic coma), 070.54 (chronic hepatitis C with no mention of hepatic coma), 070.70 (unspecified viral hepatitis C without hepatic coma), 070.71 (unspecified viral hepatitis C with hepatic coma).

The studied variables were: year of admission (2004-2013), sex (male/female), age, diagnosis (acute, chronic or unspecified hepatitis C) and days hospitalized. Co-morbidity with HIV has been studied (ICD-9-CM: 042, for Human immunodeficiency virus [HIV] disease and V08, for asymptomatic HIV). The database was cleaned to remove any potential duplicates of hospitalizations. The entries without complete values for age and sex were removed, as well as those entries with extreme values – using a refined average stay. From the initial 357,731 entries, 1.6% was eliminated and a total of 351,996 have been analysed.

For the computation of the rates we have used the population estimations of the studied period provided by the Spanish National Statistics Institute. [24]. For the purposes of standardization, the European population has been used [25].

### Statistical analysis

A descriptive analysis was carried out, and in order to compare the qualitative variables Pearson’s chi-squared test was used. To compare the quantitative values, Student’s *t* technique was used for normal distributions, and Mann-Whitney’s *U,* as a non-parametric test, for non-normal distributions. Values of *p* < 0.05 were considered statistically significant.

For the statistical analysis we have used IBM-SPSS ver. 22 and for the trend-analysis, Jointpoint Regression Program 4.2.0.2., which, through Poisson regression models, allows the calculation of Annual Percent Change (APC) and the estimation of trend change points.

## Results

Our study provides an analysis of 351,996 hospitalizations over a 10-year period, which equates to approximately 35,000 per year.

Table 1 shows the distribution of hospitalizations for the studied period, by sex, year and diagnostic category.

**Table 1:** Hepatitis C Hospitalizations. Distribution by diagnostic category, sex and year. CMBD Spain 2004-2013

	Acute			Chronic			Unspecified			Total HC		
Year	Men	Women	Both	Men	Women	Both	Men	Women	Both	Men	Women	Both
2004	1041	626	1667	19,527	11,077	30,604	0	0	0	20,568	11,703	32,271
2005	958	589	1547	19,752	11,023	30,775	0	0	0	20,710	11,612	32,322
2006	575	332	907	18,628	10,411	29,039	1081	564	1645	20,284	11,307	31,591
2007	481	247	728	18,883	10,535	29,418	1430	761	2191	20,794	11,543	32,337
2008	520	291	811	19,902	11,468	31,370	1322	771	2093	21,744	12,530	34,274
2009	306	203	509	21,274	12,274	33,548	1488	895	2383	23,068	13,372	36,440
2010	352	194	546	21,873	12,320	34,193	1489	829	2318	23,714	13,343	37,057
2011	294	209	503	22,088	12,101	34,189	1646	954	2600	24,028	13,264	37,292
2012	296	197	493	22,295	12,561	34,856	1750	950	2700	24,341	13,708	38,049
2013	287	163	450	23,843	13,350	37,193	1757	963	2720	25,887	14,476	40,363
Total	5110	3051	8161	208,065	117,120	325,185	11,963	6687	18,650	225,138	126,858	351,996

During the 10 years studied (2004-2013) a total of 351,996 hepatitis C hospitalizations were recorded (64% men; 36% women *p* = 0.031) with a 1.7 ratio of men to women. By diagnostic category, the cases are distributed between acute hepatitis (8161; 2.3%), chronic hepatitis (325,185; 92.4%) and unspecified hepatitis (18,650; 5.3%), maintaining the same ratios between men and women.

**Fig. 1:**
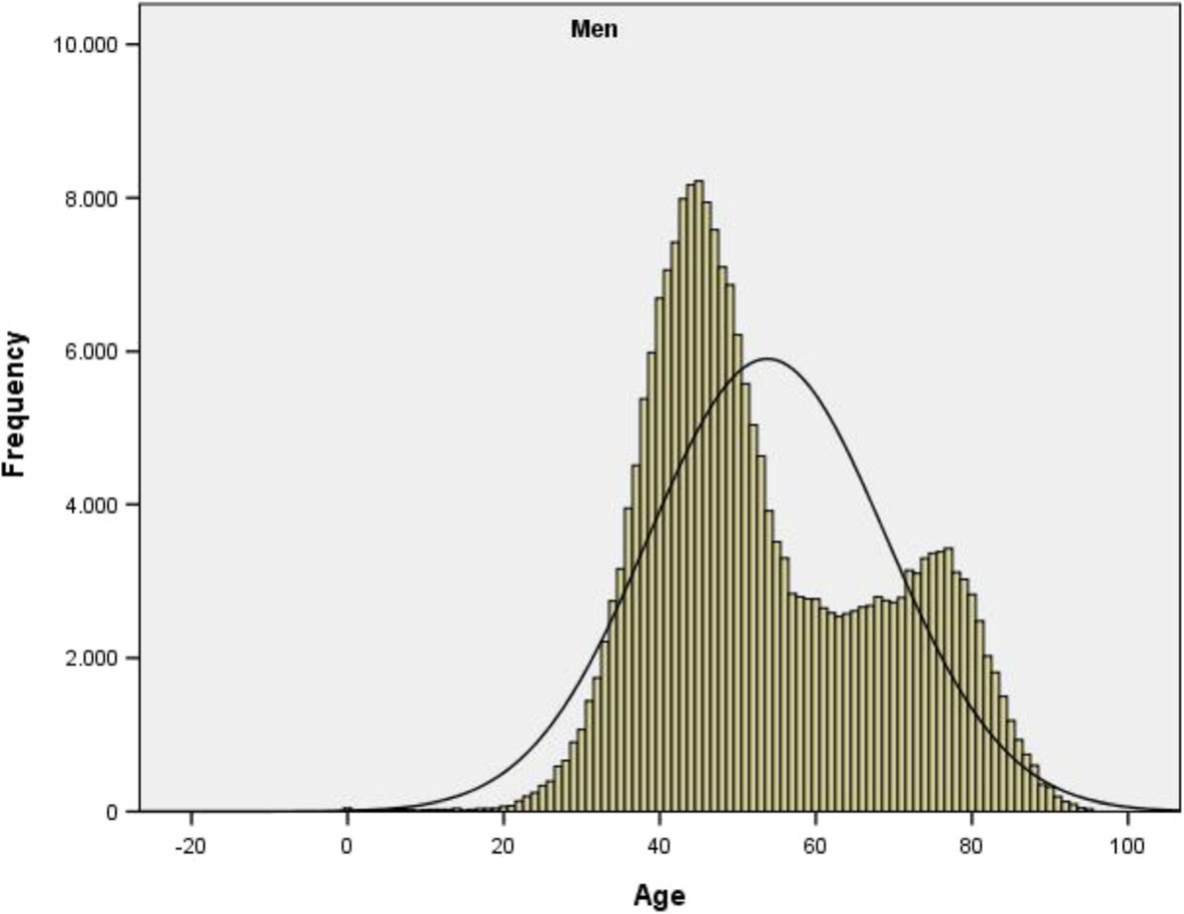
Hepatitis C Hospitalizations. Histogram of age distribution frequency. CMBD Spain 2004-2013. Men. Note. p*value < 0.05 significant value for the mean differences in age by sex, Mann-Whitney’s U *p* < 0.001

**Fig. 2:**
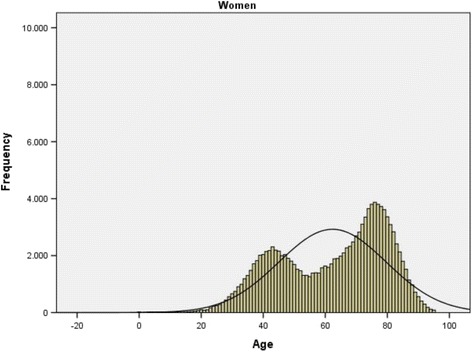
Hepatitis C hospitalizations. Histogram of age distribution frequency. CMBD Spain 2004-2013. Women. Note. p*value < 0.05 significant value for the mean differences in age by sex, Mann-Whitney’s U *p* < 0.001

Age presents a bimodal distribution (Figs. 1 and 2, Table 2). The age group with the highest frequency in men is between 40 and 49 years (N 75025; 33.35%) with a smaller peak in the 70–79 age group (N 31363; 13.90%). In women the most frequent age group is 70–79 years (N 34439; 27.10%) with a smaller peak in the 40–49 age groups (N 20752, 16.40%).

**Table 2:** Frequency distribution by age group. Hepatitis C Hospitalizations. CMBD Spain 2004-2013

	Men		Women	
Age	N	%	*N*	%
<1	44	.0	27	.0
1_4	50	.0	51	.0
5_9	107	.0	103	.1
10_14	142	.1	83	.1
15_19	154	.1	201	.2
20_24	704	.3	668	.5
25_29	2866	1.3	1883	1.5
30_34	9204	4.1	4491	3.5
35_39	22,985	10.2	8340	6.6
40_44	37,320	16.6	10,854	8.6
45_49	37,705	16.7	9898	7.8
50_54	25,379	11.3	7297	5.8
55_59	15,224	6.8	7007	5.5
60_64	13,132	5.8	8723	6.9
65_69	13,513	6.0	11,158	8.8
70_74	15,051	6.7	15,628	12.3
75_79	16,312	7.2	18,811	14.8
80_84	10,651	4.7	13,833	10.9
85+	4595	2.0	7802	6.2
Total	225,138	100.0	126,858	100.0

Highest frequency in men occurs at age 45 and in women at 76 years. The median in men is 50 (interquartile range: 42–66) and in women is 66 (interquartile range 47–77). These differences are statistically significant *p* < 0.001.

A study of co-morbidity highlights that 22.8% of hospitalizations for hepatitis C also presented with HIV and 14.7% presented with opioid addiction. Both are more frequent in men than women *p* < 0.0001 (Table 3).

**Table 3:** Hepatitis C Hospitalizations. Distribution of comorbidity with HIV and opioid dependency by age and sex. CMBD Spain 2004-2013

		Frequency	%	p*	Mean Age	SD	P**
HIV	Men	61,517	17.4		43.28	6.78	
	Women	18,882	5.4		41.99	6.93	
	Total	80,399	22.8	*p* < 0.0001	42.98	6.84	*p* < 0.0001
Opioid dependency	Men	41,395	11.7		42.22	6.68	
	Women	10,397	3.0		40.60	6.74	
	Total	51,792	14.7	*p* < 0.0001	41.90	6.72	*p* < 0.0001

p* <0.05 significant value for the difference between percentages for each sex – chi-square/χ^2^

p** <0.05 significant value for the difference between mean ages by sex – Student’s *t*

**Fig. 3:**
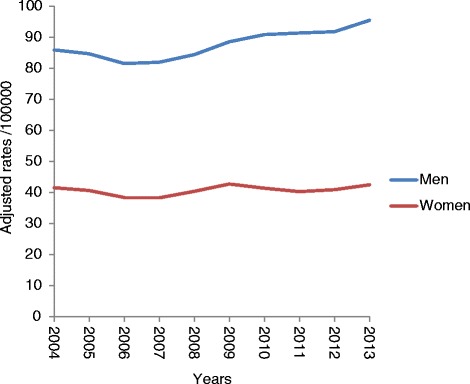
Hepatitis C Hospitalizations. Evolution of hospitalization rates according to sex. CMBD Spain 2004-2013. Note. Adjusted rates per 100,000 inhabitants for the European standard population. Poisson Regression p for trend is not statistically significant Men *p* > 0.05 Women *p* > 0.05

Hospitalization rates are shown in Fig. 3. They present a slight upward trend, which is statistical not significant, for both men and women – although some change-points can be observed. The trend for men is for an APC of 1.02 (*p* > 0.05) with a change-point in 2010; for women the APC is 0.81 (*p* > 0.05) with a change-point in 2007.

## Discussion

To the best of our knowledge, this is the first study of hepatitis C hospitalizations at a national level during a recent 10-year period. The results show a high rate of chronicity, with over 90% of cases, and significant differences for sex and age in the epidemiological pattern of the disease.

Of those admitted with this disease 64% are men and 36% are women, although the proportions of hepatitis C types remain similar for both sexes.

When we compare our data with that of the surveillance system of ECDC, we can see that there is a 1.9 ratio of men to women in European cases [2], while in our study it was a little less: 1.7. The majority of the cases recorded in Europe are classified as “unspecified hepatitis”, due to the difficulties in classifying correctly, although it is assumed that the majority are chronic hepatitis, as found in our study. The most numerous group in the European study were 25 to 44 year-olds, somewhat younger than in our study, in which the 40–49 age range in men and 70–79 age range in women were the most numerous.

In our study the age of hospitalization presents a bimodal distribution in men and women with a reversed image with a most frequent age group 40-49 years in men opposite to 70-79 years in women.

The bimodal distribution suggests that two populations with different patterns of the disease can be observed in these findings, a younger population in which men predominate and an older population in which women predominate. This distribution may indicate that the populations have been subjected to different risk factors in the acquisition of the disease, something which other studies have already noted [3]. On one hand, hepatitis C principally affects young men, and in this group the prevalence of co-infection with HIV and that of opioid dependence is much higher than for women. According to a number of studies, the rate of hepatitis C infection in people with HIV is close to 30% [26]. For populations of injection drug users there is a substantial range of infection between 21 and 86% [27, 28]. On the other, for the predominantly more advanced ages in which infection in women is found, it is probable that the infections were a consequence of nosocomial infections in the past, as a result of blood transfusions before 1990 or the use of unsterilized syringes before 1975.

A possible hypothesis is that in the past women could have been subjected to an additional risk-factor, as pregnancy and birth admissions are principal causes of hospitalization. Some studies estimate that nosocomial transmission accounts for 15–25% of cases [29, 30].

The data of hospitalizations in our study are consistent with those found in another study carried out in Spain in which more than 3000 serum samples of patients were analyzed and differences were found in the genotypes related to the age cohort, with differences between those born before and after 1950 being found [31].

Although the trend in women is less noticeable than that in men, as is reflected by the percentage of annual change, we have identified a point of inflection or change in both (in 2010 for men and 2007 for women). Therefore, with less effect there is also a slight increasing trend.

This slight upward trend in hospitalization rates adjusted for age for hepatitis C goes contrary to the trend for general hospital admissions, which have declined during the last few years. In 2013 there was a decrease of 0.47% with respect to 2012 [19], more prominent in women – owing to a decrease in the number of consultations for pregnancy, delivery and puerperium.

However, it is important to highlight a number of limitations with the use of the CMBD related to the fact that it was created for hospital management and therefore its applications in epidemiology can be limited. As CMBD data are anonymous, it is impossible to identify hepatitis C cases being hospitalized more than once for the duration of the disease. We have taken into account new hospitalizations and readmissions, so all hospitalizations are included. Therefore the results should be taken with caution and the number of patients should not be inferred, because in that case it would be somewhat overestimated. We think that this limitation does not affect the disease trend and in the absence of direct incidence data is a good proxy for the behavior of the disease. And in the other hand, the information is easily accessible and the data cover the whole Spanish population, this means that the epidemiological information refers to the whole hospitalized Spanish population and it covers a period of 10 years.

Predictive mathematical models for hepatitis C indicate that, with the current treatment rate, the total number of viral hepatitis C infections could decrease or remain stable. Nevertheless, the number of patients with chronic hepatitis will tend to increase, with a consequent increase in mortality, morbidity and their associated costs. Therefore, alternative strategies are required [32].

The challenge now is to diagnose the unknown cases of hepatitis C, as it is estimated that only 35% of cases have been diagnosed [33].

## Conclusions

Hospitalizations for hepatitis C entail a high level of chronicity, and they occur in two distinct patterns for women and men, for whom different age groups are affected. It is our view that the CMBD can be a useful tool to assess the scale of the problem and evaluate trends in hepatitis C. The knowledge of the epidemiological pattern of the disease is of considerable use to help orient health policy.

## Abbreviations

APC: Annual Percent Change
CMBD: Hospital Discharge Records Database
DALYs: Disability-adjusted-life-years
ECDC: European Centre for Disease Prevention and Control
HIV: Human immunodeficiency virus
ICD-10: International Classification of Diseases, Tenth Revision
ICD-9-CM: International Classification of Diseases, Ninth Revision, Clinical Modification
WHO: World Health Organization
